# Lack of Systematic Topographic Difference between Attention and Reasoning Beta Correlates

**DOI:** 10.1371/journal.pone.0059595

**Published:** 2013-03-27

**Authors:** Luis F. H. Basile, João R. Sato, Milkes Y. Alvarenga, Nelson Henrique, Henrique A. Pasquini, William Alfenas, Sérgio Machado, Bruna Velasques, Pedro Ribeiro, Roberto Piedade, Renato Anghinah, Renato T. Ramos

**Affiliations:** 1 Laboratory of Psychophysiology, Faculdade da Saúde, UMESP, São Paulo, Brazil; 2 Division of Neurosurgery, University of São Paulo Medical School, São Paulo, Brazil; 3 Department of Psychiatry, Federal University of Rio de Janeiro, Rio de Janeiro, Brazil; 4 Department of Psychiatry, University of São Paulo Medical School, São Paulo, Brazil; 5 Department of Neurology, University of São Paulo Medical School, São Paulo, Brazil; 6 Universidade São Judas Tadeu, São Paulo, Brazil; 7 Center of Mathematics, Computation and Cognition, Federal University of ABC, Santo André, Brazil; University of Rome, Italy

## Abstract

Based on previous evidence for individual-specific sets of cortical areas active during simple attention tasks, in this work we intended to perform *within individual* comparisons of task-induced beta oscillations between visual attention and a reasoning task. Since beta induced oscillations are not time-locked to task events and were first observed by Fourier transforms, in order to analyze the cortical topography of attention induced beta activity, we have previously computed corrected-latency averages based on spontaneous peaks of band-pass filtered epochs. We then used Independent Component Analysis (ICA) only to single out the significant portion of averaged data, above noise levels. In the present work ICA served as the main, exhaustive means for decomposing beta activity in both tasks, using 128-channel EEG data from 24 subjects. Given the previous observed similarity between tasks by visual inspection and by simple descriptive statistics, we now intended another approach: to quantify how much each ICA component obtained in one task could be explained by a linear combination of the topographic patterns from the other task in each individual. Our hypothesis was that the major psychological difference between tasks would not be reflected as important topographic differences within individuals. Results confirmed the high topographic similarity between attention and reasoning beta correlates in that few components in each individual were not satisfactorily explained by the complementary task, and if those could be considered “task-specific”, their scalp distribution and estimated cortical sources were not common across subjects. These findings, along with those from fMRI studies preserving individual data and conventional neuropsychological and neurosurgical observations, are discussed in support of a new functional localization hypothesis: individuals use largely different sets of cortical association areas to perform a given task, but those individual sets do not change importantly across tasks that differ in major psychological processes.

## Introduction

The main finding of a series of studies in our Laboratory, both on the cortical topography of Slow Potentials (SPs) and task-induced beta oscillations, has been the high inter-individual variability in the sets of cortical areas electrically active mainly during expecting attention [1]–[4]. This kind of variability in cortical distribution of non-sensory-motor activity, initially encountering some skepticism from the scientific community due to the challenge of strict cortical functional localization views, is now becoming increasingly accepted. Its acknowledgement has even motivated the development of new approaches to present and interpret fMRI results [5]–[7]. In particular, it suggests the inadequacy of the use of *spatial* averaging of task-related physiological changes across subjects [8]–[14], in some cases even for sensory activity [11], [12]. For this reason, our main intention in the present work was to devise means of comparing task-related results *within-individuals*.

Beta oscillatory activity, traditionally associated with overall arousal levels [15], may now be considered as belonging to a beta-gamma physiological continuum, following the convergence of many years of results between groups of investigators, mainly centered on the electrical properties of thalamocortical cells across vertebrates, which lead to a seminal review work [16]. We believe that the increasing interest in this frequency range stems from the fact that it displays the space-time dimensions compatible with long range cortico-cortical communication, in particular synchronization between areas [17], [18]. We have recently been able to analyze the cortical topographical distribution of beta activity, when increased/induced by simple attention tasks, due to new methodological approaches: Since induced beta activity is not time-locked to task events, it was originally observed only as ‘power’ changes, and its source modeling by familiar algorithms made possible only when we started to compute corrected-latency (spontaneous peak-centered) averaging [3]. However, in an analogous way to the limitation of conventional event-related averages to stimulus-locked activity, our corrected-latency averages appear to be limited to phase-locking between sources of oscillations. Therefore, following the more adequate methodological suggestions by Onton and colleagues [19], in the present work we decided to re-compute peak-centered epochs but *avoiding averaging* afterwards, and apply Independent Component Analysis (ICA) to large sets of concatenated epochs. This approach, a more proper use of ICA than our previous one (merely to extract significant signal components from estimated noise), became only recently possible due to the large computational resource requirements involved.

In a recent study [4], we came to a psychophysiological conclusion as unsettling as the inter-individual variability itself: The persistency on *within*-individual task-related activity comparisons, lead us to start noticing a major similarity between tasks and possibly minor overall significance of differences. We believe that a reconsideration of differences found across tasks in large number of studies, and an increased caution in the philosophical interpretations of statistical differences in biological experimental studies of the kind should take place in the near future. In that last study, by attempting to decide whether possibly found inter-modal (audio-visual) differences in beta activity could be localized and compared, we concluded that the shift of attention to the auditory modality did not correspond to any topographical change systematic across subjects, as analyzed by simple descriptive statistics. Moreover, the possibility remains that the differences found in that, as a single example among a possible multitude of studies, belong within an ‘experimental noise’ range loosely considered.

Since we could not rule out the effect of inter-individual variability in some our previous studies as stemming from the complexity of the task used [1], [20], we have decided to use the same simple visual attention task for many years as our comparison standard. In the present study, we intended to perform the within-individual comparisons between that simple attention task and one in which a major engagement of psychological processes were involved: a reasoning task adapted from standardized psychological tests. Given that in pilot testing we observed a major similarity between tasks in independent component patterns, on the methodological end, besides the proper use of ICA, we devised a novel approach: the application of multiple linear regression models to quantify how much each component obtained from one task could be explained by the components obtained from the other task. Our hypothesis of the present work was that the major psychological difference between tasks would not be reflected as important topographic differences, i.e., similar sets of active areas would be active in both tasks *in each subject*. But in case a set of components were to be found not explained by the complementary task, we intended to perform source localization to illustrate whether such putative ‘task-exclusive’ components were topographically systematic across subjects.

## Methods

### Subjects

Thirty healthy individuals with normal vision and hearing, 18 male and 12 female, participated in the study. They ranged in age between 20 and 50 years, with no history of drug or alcohol abuse, and no current drug treatment. All subjects signed consent forms specific to this study, approved by the Ethics Committee of the University (Ethics Committee of Universidade Metodista).

### Stimuli and Task

A commercial computer program (Stim, Neurosoft Inc.) controlled all aspects of the tasks. Visual stimuli composing the cue-target pairs (S1–S2) of the attention task consisted of small rectangles (eccentricity ±0.8°, S1∶100 ms duration, S2∶33 ms; white background). In half of the trials, the S2 rectangle contained a grey circle – the task target - with ±0.3° of eccentricity. A masking stimulus had the same grey level as the target (a ‘checkerboard’ grey and white square composed by one-by-one pixel size squares), and was continuously present, along with the fixation point, except during S1 and S2 presentation. S1 was followed by S2, with onsets *separated in time* by 1.6 seconds. The ITI was variable, ranging from 2.3 to 5 seconds. We instructed the subjects that a rectangle would be presented to indicate that 1.6 seconds later it would flash again but quickly, containing or not the target circle. The subject decided whether there was a target inside the S2 rectangle, and indicated presence of the target by pressing the right button with the right thumb or absence of the target by pressing the left button with the left thumb. We explicitly deemphasized reaction time in the instructions and measured performance by the percent correct trials, from the total of 96 trials comprising the task. An eye fixation dot was continually present on the center of the screen, as well as a stimulus-masking background, to prevent after-images.

The reasoning task consisted of 48 questions and 48 corresponding answers. Each question stayed on the screen until the subject decided to check the answer, by pushing either button. If the answer was considered correct, the subject should press the right button with the right thumb, if incorrect the left button with the left thumb. Actually correct answers followed 50% of trials in random order. The questions were divided in 3 blocks of verbal-logical, numerical-logical and visual-abstract-induction tests, adapted to computer presentation from standardized psychological tests (HTM- Brazilian adaptation of General Mental Ability Test by Santarosa, and Raven’s progressive matrices test [21], [22]).

### EEG Recording and Acquisition of MRIs

We used a fast Ag/AgCl electrode positioning system consisting of an extended 10–20 system, in a 128-channel montage (Quik-Cell, Compumedics Limited), and an impedance-reducing saline solution which restricted the need for skin abrasion to the reference and ground electrode regions. Impedances usually remained below 5 kOhms, and unstable channels were eliminated from the analysis. To know the actual scalp sampling or distribution of electrodes in each individual with respect to the nervous system, we used a digitizer (Polhemus®) to record actual electrode positions with respect to each subject’s fiduciary points: nasion and preauricular points. After co-registration with individual MRIs, the recorded coordinates were used for realistic 3D mapping onto MRI segmented skin models, and later used to set up the source reconstruction equations (distances between each electrode and and each dipole supporting point). Two bipolar channels, out of the 124-channels in the montage were used for recording both horizontal (HEOG) and vertical electro-oculograms (VEOG). Left mastoid served as reference only for data collection (common average reference was used for source modeling) and a frontal midline electrode was used as the ground. We used 128-channel DC amplifiers (Synamps 2, Neuroscan- Compumedics) for data collection and the Scan 4.5 software package for initial data processing. The filter settings for acquisition were from DC to 200 Hz, and the digitization rate was 1000 Hz. The EEG was collected continuously, and task-related epochs spanned the interval from 300 ms before S1 to 400 ms after S2 in the visual attention task, and from 2400 ms *previous* to button press to 200 ms after that, in the reasoning task. We chose this time window for the reasoning task due to the expected high variability in reaction time (confirmed to be roughly of 4 seconds, with a standard deviation above 2 seconds, by pilot testing), and to the supposition that the critical processes of reasoning would more regularly precede the decision and its motor implications (we used the window between 2000 and 500 ms before button press for the remaining analysis; see below). Baseline was defined from the first 300 ms of either type of epochs. Epoch elimination was performed visually for eye movements and muscle artifacts, and then automatically: visual inspection served to eliminate epochs containing other artifacts spread to many electrodes, such as head/cable movements. Isolated electrodes presenting frequent transient electronic noise were also eliminated visually, and additional electrode elimination was dependent on the first rounds of ICA computations. Eye blinks were removed from the continuous EEG recordings by PCA filtering, prior to the computation of epochs. We used PCA for this purpose because eye-blinks are spatially stable, and our software performs this individual-specific cleaning in a simple and straightforward way: two or three first PCA components of a short time window explaining a blink define a filter to be applied to the whole continuous data (whereas the ICA available in our package, in principle applicable to this purpose, is inadequate for this use due to computational limitations).

MRIs were obtained by a 1.5 Tesla GE machine, model Horizon LX. Image sets consisted of 124 T1-weighed saggital images of 256 by 256 pixels, spaced by 1.5 mm. Acquisition parameters were: standard echo sequence, 3D, fast SPGE, two excitations, RT = 6.6 ms, ET = 1.6 ms, flip angle of 15 degrees, F.O.V = 26×26 cm. Total acquisition time was around 8 minutes.

### Frequency-Time Analysis

After artifact rejection, the signal from each channel was spectrally analyzed by means of a Short Time Fourier Transform (STFT), to obtain frequency-time charts of the induced spectrum from both tasks, from each individual, to confirm the attention induced beta and to verify whether the reasoning task was also accompanied by beta power changes. To obtain the induced power spectrum [23], the time-frequency decomposition was made for each electrode and each trial, from DC to 100 Hz, and the resulting charts were then averaged, both for each electrode and across electrodes. The decomposition was computed twice on the EEG tapered by two sliding Hamming windows, 400 points in size for inspection of frequencies over 30 Hz, and 1000 points for lower frequencies, with a temporal resolution (window displacement) of 10 points, and a frequency resolution of 8192 points. Then, we normalized the average power for each electrode to obtain Z-scores of increments or decrements in each frequency bin with respect to the power in the same frequency during the 300 ms baseline (<P_j_> = (P_j_−µ_j_)/σ_j_; given P_j_ = spectral power at each time point in electrode j, µ_j_ and σ_j_ are the mean and standard deviation, respectively, of the average power during the baseline for the electrode). Among the various available methods for frequency-time analysis, we chose STFT because we are familiar with the appearance of its results, and dispose of a fairly large database from previous studies computed in this fashion. Moreover, we used it exclusively to confirm the presence of beta induced activity in each subject and task, and its results had no implications to the main analysis of the present study.

### Re-computation of Peak Centered Epochs and Independent Component Analysis

Original EEG epochs were filtered between 15 and 30 Hz: Butterworth, 96 dB roll-off. The resulting filtered epochs were then processed by an algorithm for searching the peaks of bursts within the task-time windows of interest. Filtered epochs were thus cut again starting from positive voltage peaks (automatically searched in the stimulus-expecting time window for the attention task 500 to 1600 ms after S1, and between 2000 and 500 ms before button-press for the reasoning task), resulting in new epochs, ranging from 100 ms before to 100 ms after the peaks. As previously, we used each channel in the search for peaks, thus leading to a large number of new epochs: number of good channels multiplied by the number of original good epochs. The new epochs were shuffled randomically so that any portion of their concatenated set became representative. This was confirmed by pilot testing, which also served to determine the maximum computational capacity available in our laboratory for ICA and particularly ICA filtering: with a peak of 2.7 Gigabytes of RAM use during ICA filtering, we were able to analyze data matrices corresponding to 350 to 500 thousand time points, safely above the recommendation of Onton and colleagues [19] (20×N^2^−N = number of good channels - time points), which in our case would mean between 160 and 290 thousand time points.

Our pilot testing simultaneously determined the maximum number (by trial and error, with successful ICA convergence), of 10 independent components obtainable for the filtered data, by the fast-ICA algorithm included in the commercial software package used in our analysis (Curry6, Compumedics Limited). We are aware of other types of software capable of computing larger number of components, but our algorithm computes this maximum of 10 components, which we consider a reasonable number, since the data are filtered in a single band. As mentioned above, the first two or three ICA rounds served to eliminate additional electrodes manifesting transient artifacts, and to determine whether the data from each subject resulted in the largest possible number of components of supposed brain origin (remaining after the elimination of obvious or suspected muscular components): components representing muscular activity (sharp polarity reversals, with voltage extrema comprising immediately neighboring frontal, temporal or occipital electrodes) were not included in the analysis. Since muscular activity is not passible of PCA filtering, which we also tried during the pilot tests, subjects presenting a major ‘contamination’ by muscle activity were eliminated from analysis. Six subjects were thus eliminated, 3 for this reason and 3 for excessive electronic noise). Besides the obvious cases to visual inspection (as performed by other groups, e.g., [19], [24]), suspected muscular components (with voltage extrema spreading a little beyond immediately neighbor electrodes), were spectrally analysed: the plateau shaped power distribution reaching very high frequencies were considered of muscular origin. Finally, those components not considered artifactual, among the 10 remaining after a third ICA step, were considered of brain origin. We eliminated an average of 24 channels in the remaining group of 24 subjects, on whose data the full analysis was performed.

### Statistics of ICA Results

For each subject, a multiple linear regression was independently modeled for each of the 10 ICA channel coefficients of one task (as the response variable), considering the 10 ICA coefficients of the other task as predictor variables. Components of suspected muscular or electronic artifactual origin were not considered in the analysis. The basic idea of this analysis is to evaluate whether the two tasks have spatially similar components (or linear combinations between them). We considered components highly similar between tasks, i.e., statistically explained by the complementary task, when the resulting adjusted R2 values of the multiple regression were above the cutoff value of 0.85. In other words, we considered that a component was similar between the two tasks if 85% (or above) of its variance (across channels) could be explained using the components of the other task. The percentage of similar components for each task and subject were tabulated for the global consideration of results.

### Intracranial Source Reconstruction

The independent components from the reasoning task that eventually would be found not to be explained by the attention task were exported by ICA filtering of the original data, for source reconstruction, to test for a possible common origin across subjects. MRI sets were linearly interpolated to create 3-dimensional images, and semi-automatic algorithms based on pixel intensity bands served to reconstruct the various tissues of interest. A Boundary Element Model (BEM) of the head compartments was implemented, by triangulation of collections of points supported by the skin, skull and cerebrospinal fluid (internal skull) surfaces. Mean triangle edge lengths for the BEM surfaces were, respectively, 8, 7 and 5 mm. Fixed conductivities were attributed to the regions enclosed by those surfaces, respectively, 0.33, 0.0042 and 0.33 S/m. Finally, a reconstructed brain surface, with mean triangle side of 2 mm, served as the model for dipole positions. Individually measured electrode positions were used, and finely adjusted onto the skin’s surface modeled from the MRIs (2 mm mean triangle side). The detailed description of the assumptions and methods used by the “Curry 4.6” software for MRI processing and source reconstruction may be found elsewhere (e.g., [25]–[27]). The analysis program then calculated the lead field matrix that represents the coefficients of the set of equations which translate the data space (SNR values in the set of channels per time point) into the model space (the thousands of dipole supporting points). The source reconstruction method itself was sLORETA, with data Lp norm = 2, also part of the Curry6 software package.

## Results

### Task Performance and ICA Results

All subjects reported that performance was relatively easy during the attention task, provided that they were strongly attending during the critical time of S2 presentation. Six subjects were eliminated from the study, three for excessive temporal and frontal muscle activity, and three due to excessive periods of electrode instability or electronic noise mainly from electrode cable movements. Further analysis to be presently reported was thus performed on data from 24 subjects (16 male and 8 female). The overall average performance in the attention task was 90.2% correct responses (standard deviation 13.2%) and 82.1% in the reasoning task (standard deviation 9.5%). This difference was statistically significant (t-test, p = 0.02).

**Figure 1:**
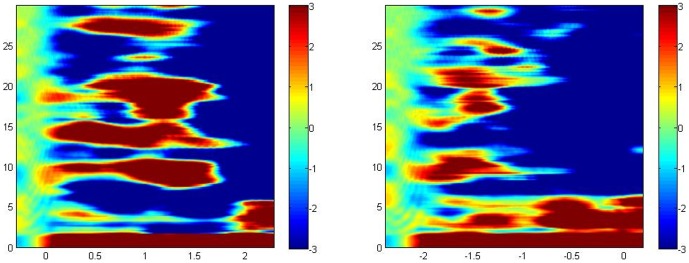
Examples of time-frequency plots, showing the total task-related power changes transformed into z-scores (scale at right of each figure); (left) attention task, (right) reasoning task. At bottom, task time in seconds, mostly negative during the reasoning task, with respect to button pressing. At left of each figure, frequency in Hz.

As previously observed, all subjects presented increased beta activity in two or more sub-bands during the attention task, typically peaking around 1 sec after S1 presentation. As a new result, all subjects also presented beta induced activity preceding the decision/response time of the reasoning task, in similar frequency sub-bands, typically peaking around 1.5 sec before button press. Figure 1 presents the z-transformed frequency-time plot of task-related power changes from the attention and reasoning tasks, respectively, from one example subject.

**Figure 2:**
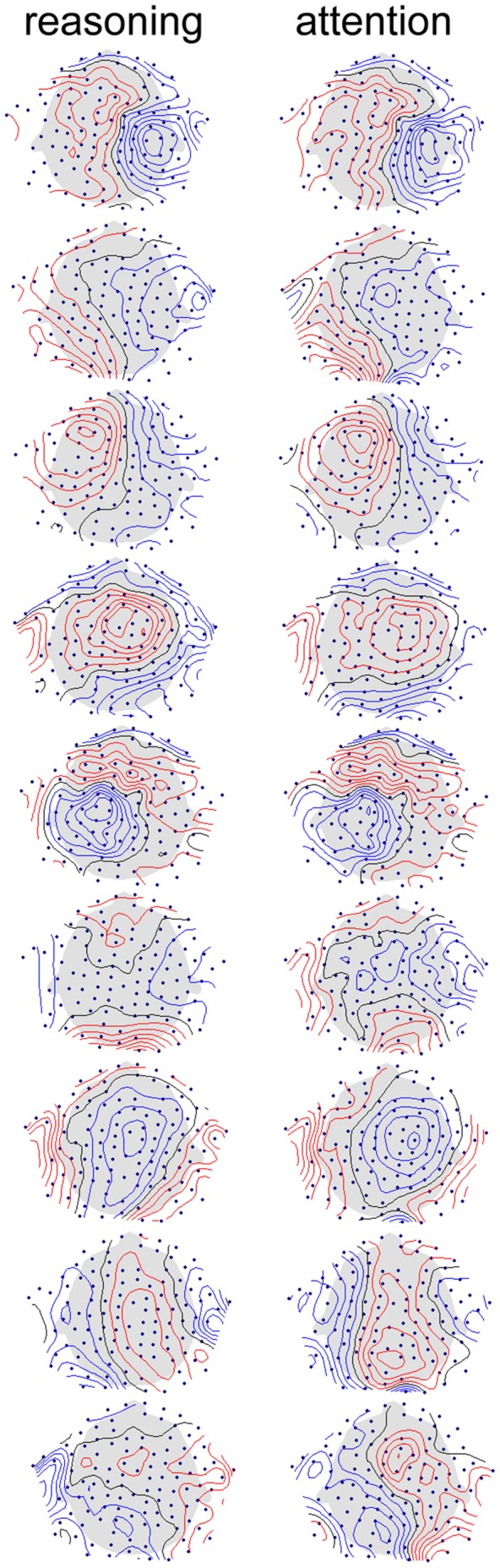
Example set of ICA topographic patterns obtained for one subject, after decomposition of beta activity during both tasks. Colors indicate opposite polarity.

After the first ICA rounds and additional electrode elimination, the remaining electrode sets averaged 104±10 in number. The induced beta decomposition resulted in good, typical cortically originated topographic patterns, averaging 6±2 components across subjects, for both attention and reasoning tasks. As expected from the work of Onton and colleagues [19], the patterns were relatively simple (with few extrema, many times of dipolar appearance) as compared with our experience on corrected latency averaged patterns. Figure 2 shows ICA patterns from an example subject who presented 9 good components, for both tasks.

### Statistics of ICA Results

The adjusted R2 values for each component for the two subjects presenting 9 good components for each task are shown in Tables 1 and 2 (values from subject represented in table 1 corresponds to the maps presented in figure 2). The average percentage of explained components (adjusted R2 above 0.85) was 89.4% (±15.7%) for the attention task and 86.3% (±18.0%) for the reasoning task. This difference was not statistically significant (t-test, p = 0.19). The overall average values of adjusted R2 for all ICA components across subjects were 0.92 (±0.07) for the attention task, and 0.92 (±0.08) for the reasoning task. If we consider only the explained components, the averages were also virtually identical, 0.944 (±0.037) and 0.941 (±0.039) for attention and reasoning tasks, respectively. If we consider only the 46 unexplained components across all subjects, the average adjusted R2 values are still high, 0.76 (±0.09) and 0.73 (±0.13) for attention and reasoning tasks, respectively. Ten of the twenty three subjects presented all components from the reasoning task explainable by the attention task components, and thirteen of the subjects presented all components from the attention task explainable by the reasoning task components.

**Table 1:** Multiple Linear Regression R2 Values.

0.9915650	0.9868824
0.9858277	0.9824556
0.9782438	0.9903828
0.9859431	0.9687755
0.9264988	0.9691128
0.9712228	0.9768545
0.8675914	0.9757552
0.9744756	0.9433747
0.9046056	0.9652627

Examples of adjusted R2 linear regression values obtained for the subject whose ICA maps are shown in figure 2, who presented 9 good ICA components for beta activity. At left, adjusted R2 values for the reasoning task components, at right, for the attention task components.

**Table 2:** Multiple Linear Regression R2 Values.

0.9820455	0.9942912
0.9763507	0.9855181
0.9935971	0.9723158
0.9787430	0.9902790
0.9876926	0.9682983
0.9780159	0.9593978
0.9728619	0.9723354
0.9585617	0.9420752
0.8937664	0.9055980

Examples of adjusted R2 linear regression values obtained for the other subject who presented 9 good ICA components for beta activity.

### Source Reconstruction and Cortical Distribution of Unexplained Components

**Figure 3:**
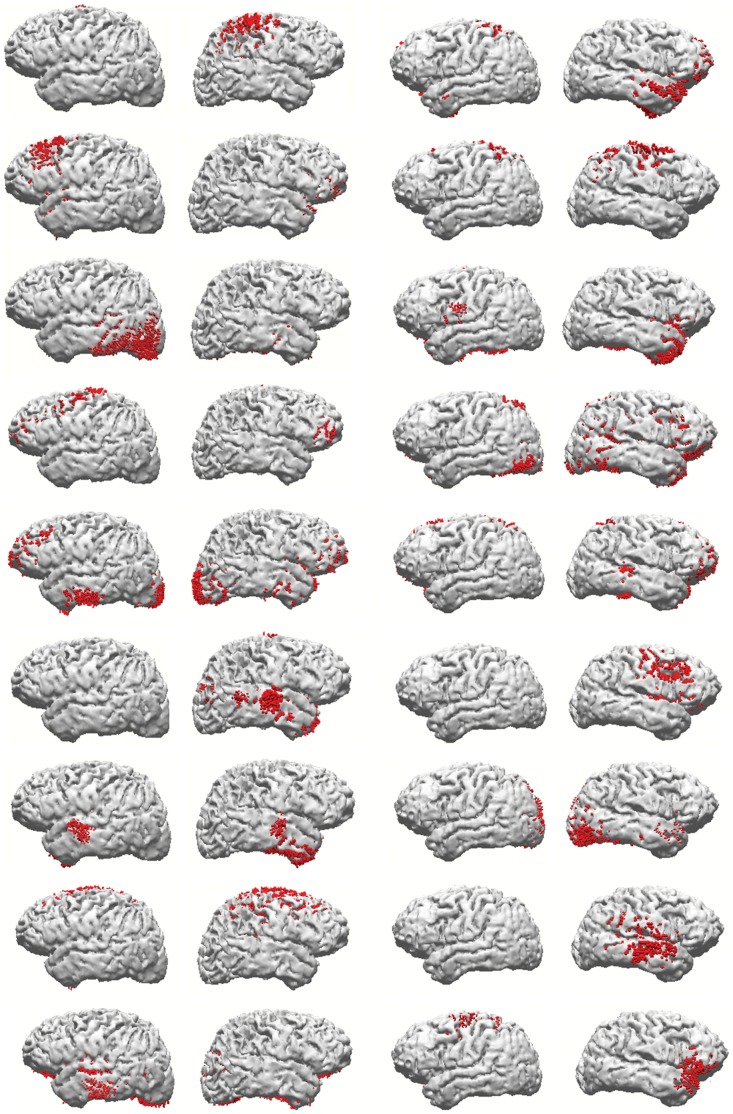
Examples of source reconstruction results obtained for the reasoning task beta activity, from the two subjects (each column) who presented 9 beta ICA patterns (sLORETA algorithm, data-Lp norm = 2; current density distribution clipped at the percentile 50 of the maximum current in each case and subject).

**Figure 4:**
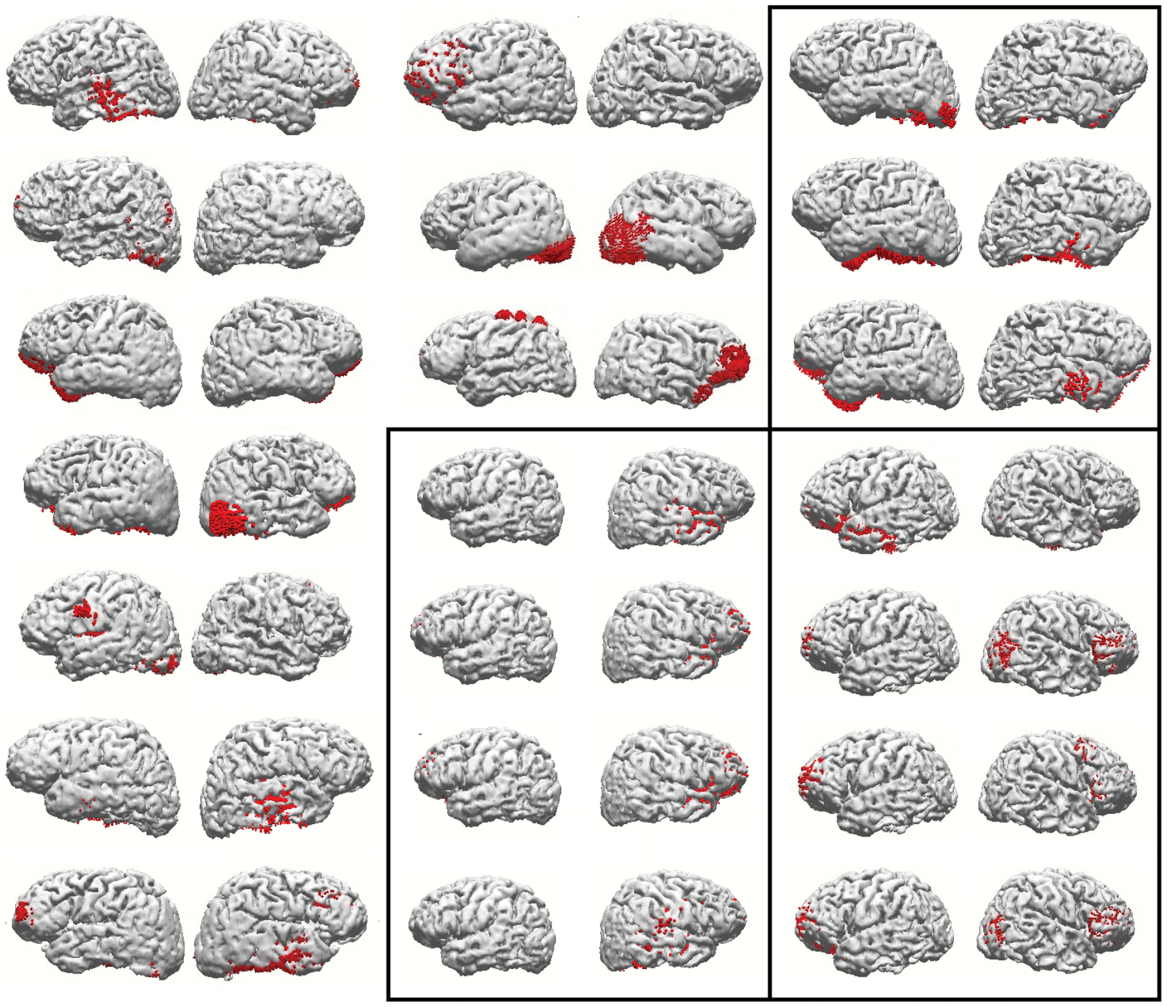
Representation of source reconstruction results (as in **figure 3**), of the reasoning task components from all subjects, that were not satisfactorily explained by the attention task. The rectangles indicate the three subjects who presented more than one unexplained component.

Figure 3 shows two examples of reconstruction results for all components from the two subjects who presented 9 good components. As we have always observed when analyzing both averaged Slow Potentials and corrected latency averaged beta activity, the sets of cortical areas estimated as sources of the present epoched data are highly variable across subjects. The figure is an example of the fact, typical of case-by-case inspection of results, that few example subjects are sufficient to show the lack of commonality in sets of active cortical areas across individuals. When sources of all components from a subject and task are taken together, they seem complex and idiosyncratic as the ICA components from averaged data with which we are familiar. However, sources of single components extracted from the present epoched data are typically more focal, but sometimes bilateral. We have also inspected all components that were not satisfactorily explained by the complementary task. Figure 4 shows reconstruction results for all components from the reasoning task that were not satisfactorily explained by the attention task components, in the thirteen individuals in whom they occurred. Those results are also sufficient to demonstrate the lack of similarity across subjects on what could be a ‘task-specific’ set of cortical areas related to the additional processes involved in reasoning, as opposed to mere visual attention.

## Discussion

### Individual Variability in Active Association Areas and Functional Localization

The high degree of inter-individual variability found in the scalp distribution and estimated cortical sources of Slow Potentials (SPs) and beta oscillations in our previous studies [1]–[4], [20] has raised the need for within-individual methods of analysis, task comparisons in particular, the main purpose of the present work. This type of variability, in the *sets of cortical areas active* across individuals, was once more found in our estimated sources of beta activity during the present visual attention and reasoning tasks: no single cortical area was seen to be active in all subjects. This variability is also met with in the unfortunately few fMRI studies that present individual data [8]–[14], some even during passive sensory stimulation [11], [12]. Nevertheless, the concern with individual variability is now an important issue in fMRI research, but typically of a different kind, regarding the extent and amplitudes of task-related changes in given brain regions, frequently pre-selected ‘regions of interest’ (ROIs). The proposed methodological improvements center in the transformations of individual data (coordinates of peak changes) to a common ‘space’, *previous to group spatial averaging* [5]–[7]. In few cases we find the concern to preserve individual data and an explicit advice against group spatial averaging [8]–[14]. The methods of analysis and interpretation in those studies would typically range from simple data tabulation to ‘fuzzy clustering’ [28] or ‘multisubject network’ [29], [30] approaches. It is still worthwhile emphasizing that group averaged spatial patterns does not match each and every individual, in that a given cortical area depicted in the pattern may show *no change at all* in some individual, and some individual may present important changes in areas not seen by group averaging or not belonging to the selected ROIs. We may mention here some examples of studies closely related to our line of research, where results appear to be more consistent across subjects, but this fundamental difference may be explained by methodological issues. Thus, some groups interested in beta activity use electrical power to be mapped instead of ICA decomposed voltage as we did [24], [31], [32]. Average power essentially “collapses” various out-of-phase (independent) sources in space, as compared to what we see by the current ICA on *unaveraged* data – which on its turn also “sees more” than our previous method of corrected latency averaging, that emphasizes phase-locked sources. Regarding sampling, in the study by Kamiński and colleagues [24], only 11 electrodes were used, and the own authors explain the lack of expected findings in the occipital region by this low resolution. Hanslmayr and colleagues [31] pool only 8 parieto-occipital electrodes out of 64 for statistical analysis. More important, in the study by Hipp and colleagues [32], the consistency for the group of the 24 subjects at first impression stems from one more example of study (as some fMRI studies mentioned above) where different individual measures (power projected to a common grid of 400 points by beamforming) are subject to group statistics on secondary measures (coherence between sources) before the process of “network identification”, thus performed on group data. Besides the different measure, of source coherence during the peri- and post-stimulus *reduction* in beta activity (that could in principle topographically differ from the prevailing stimulus expecting activity, being more stimulus/perception related), the essential point is that for that study to be comparable to ours, networks should be individually “identified”. One interesting invasive study of beta activity in monkeys [33] shows increased beta coherence between frontal and parietal cortices during a kind of expecting attention (visual search), but only four areas were sampled by electrode implantation. As previously discussed by us [3], for studies in experimental animals, especially single cell recordings, to be comparable to ours, the prevalence of cells classified by response type to tasks, i.e., their distribution in different cortical areas, with a widespread sampling, would have to be explicitly compared across a number of animals. And according to the experience of Prof. J.M. Fuster (personal communication), that distribution is also highly variable across individuals. Finally, we may cite the fMRI study on visual attention by Corbetta and colleagues [34], where even with a small number of ROIs analyzed in only 5 subjects, the table of individual results presents gaps in three subjects and two out of 5 areas. Comparability to our method in this case would mean increase in sample, consideration of all task-related raw, *unprocessed* BOLD effect distribution across the cortex in each subject, and especially a verification of how by varying the arbitrary statistical thresholds to present results would affect the *individual* patterns of BOLD effect cortical distributions.

If individual data were always taken into account, even if one single subject in each study presented no changes in some area appearing in group averaged results, the consideration of the enormous amount of studies and tasks would leave no single area as essential for the implementation of any task. This lack of one-to-one function-to-area mapping will certainly also be the case even if group spatially averaged data are considered, regarded that *unrestricted literature search* is performed either by cytoarchitectonic area or by supposed psychological function. One explicit theoretical account of the variability in cortical areas active during particular tasks, and in clinical symptomatology after lesions in common areas, an implicit commonsense fact that justifies the statistical nature of Neuropsychological results, is the ‘biological degeneracy’ concept [30]: it allows and emphasizes many-to-one function/area possible mappings.

The most solid basis for the contemporary version of localization of function, and corresponding scientific community resistance to other views, stems from the anatomy of preferential cortico-cortical connections [35]–[37], particularly developed in the case of the visual cortices [38]. It has motivated our own initial search for regional specialization of prefrontal cortical function [1], [39], [40]. By now we believe that a new hypothesis, of “individual localizationism”, becomes plausible: the consideration of two or three long-range (excitatory-excitatory) cortico-cortical synaptic steps leads one to conceive of a myriad of possible *functional* loop pathways, to be formed and possibly changed spontaneously, and given the complexity involved, in an individually idiosyncratic fashion. Of course, preferential (“one-synaptic”) pathways could, in principle, define a much expected universal functional pattern across individuals, of statistical ‘probabilistic’ nature, if unreasonably large numbers of subjects were included. This is exactly the purpose of ‘meta-analytical’ studies, but it is a fact that in spite of more than four decades of research resulting in a vast literature on task-related physiological changes, no conclusive, consensual “human brain map” encompassing cortical areas beyond the sensory-motor domains is still available. On the other hand, a most critical support for the suggested “individual-localizationist” hypothesis of association cortical function is Functional Neurosurgery. Beyond the well-recognized facts of occasionally undetectable symptoms after localized cortical lesions or resections, and the wide variability in individual ‘eloquent’ areas, for instance, intraoperative interference with cortical function by electrical stimulation is now explicitly recommended and extended to “noneloquent” areas [41]. Particularly relevant is the fact that this recommendation allows for safer tumor resections beyond visible lesion margins, with lack of functional impairments in many cases [42].

### Minor Relevance of within-individual Task-differences

Regarding the task comparison performed in the present study, it represents a new effort that may also contribute as another interesting aspect of the ‘individual localizationist’ hypothesis, and as a general advice against another major trend in research. It is an attempt to establish a quantitative foundation for the high topographical *similarity* between tasks, *within subjects*, a common observation in all of our data, from this and previous studies. Statistics applied to biological sciences is designed to test for differences. Following its regular use and mainstream search for differences, we have previously attempted to test for task-specific SP generators during verbal, pictorial and spatial visual tasks, undistinguishable by visual inspection ([1]; we have also desisted from publishing results from the detection potentials of the P300 class from the same tasks, exactly due to the lack of significant task differences). To avoid spatial group averaging, we tabulated scores of activity in estimated cortical areas, and performed group analysis on the spatially abstracted data. Although a few areas were depicted by the analysis as putatively “task-specific”, we were then already careful to state that the effect could well disappear, for instance, after sample increase, and the above comment on *unrestricted* literature searches. In an analogous way, when computing corrected-latency averages of beta oscillations and estimating their sources [3], we concluded that the absolutely major fraction of ‘task-induced’ beta activity is topographically identical to the pre-stimulus baseline beta (95% of power), thus not ‘task-specific’ but merely an increase in the ongoing activity. The putatively ‘task-specific’ much weaker component (in that case extracted as the second ICA component of the averaged data) could belong to the typical kind of result more prone to naturally vanish by lack of replicability, for instance, immerse in “experimental error” in a wide sense. In a more recent work, we explicitly compared the generators of averaged beta oscillations, between the current visual attention task and one with the same visual stimuli but with superimposed auditory stimuli to be attended [4]. Again, a very small fraction of estimated currents could be said to differ between tasks, i.e. correspond to the modality shift of attention, but with no common pattern across subjects. In the present study, some methodological improvements with respect to that study gave strength to the conclusions: 1- a completely independent blind analysis (ICA) was performed on data from each task, thus without task-data subtraction; 2- the avoidance of averaging allowed for more components to be obtained, overall, for each task, a major challenge to the similarity test, as opposed to typically two components obtained from averaged data ICA; 3- moreover, regarding the quality of components, ICA applied to epochs represents a proper use of the method, whereas the previous use of ICA on averages was simply a method to extract the most significant part of the data (above some SNR level); 4- finally, averaging emphasizes the phase-locked portion of oscillatory signals. Results from the presently used multiple linear regression modeling between task components indicated a very high topographic similarity between beta activity from the two very different tasks. Overall, very high adjusted R2 values were obtained for all components, even for those that did not pass our cutoff criterion of ‘reciprocal explainability’. Those components, not considered to be reciprocally explained between tasks, on their turn, were once more not systematic in topography across subjects. This is a new, independent indication that tasks that differ in major psychological processes may not be so different in some of their physiological correlates, depending on the way we look at the data. It is interesting to re-mention here the fMRI work by Corbetta and colleagues [34] that also supports the idea of largely common activity across supposedly very different tasks, involving visual-spatial attention, covert and even explicit saccades. Although the only band of interest in this study, beta activity should represent a most direct correlate or indicator of cortico-cortical communication ([17], [18], [43]; its physiological continuum counterpart, the gamma band, was not analyzed due to the well-known individual variability in bands and amplitudes [44]; besides the more difficult technical issues of muscle activity contamination). Experimental and modeling evidence differentiate beta and gamma in this respect, leaving gamma synchronization as a mediator of more local cortical interactions [45]. Most investigators regard beta as a correlate of attention/arousal and performance (efficient task engagement), processes so universal as to coexist with mere consciousness (thus obviously present in both of our tasks). One slightly different view, by Engel and Fries [46], holds beta activity as “signaling the status-quo”, in the motor case, of the “motor set”. We prefer the more parsimonious view held by the other cited groups [24], [31], [32] regarding beta as a correlate of attention/arousal, and suggest low beta (including the μ rhythm) to be considered a “high or motor” alpha, given its opposite behavior to higher frequency beta bands (indicating cortical ‘idling’ ([47]; in its case, of motor areas). If beta is not related to attention/arousal *per se*, it would be connected with other equally vague and universally present processes such as mental effort, task engagement or voluntary action, all concurrent with mere consciousness, to some degree; and such degree is exactly what appears to be co-modulated with beta. Finally, we consider any of those putative psychological correlates of beta perfectly compatible with its role in long-range cortico-cortical communication, i.e., they would essentially reflect such communication (along with its subcortical counterpart, undetectable by the scalp EEG methods). In any case, as stated in a previous study when all bands were analyzed, at least the main component of all frequency bands appear to have the same generators ([3] with exception of alpha-2, SPs or ‘sub-delta’, and occasionally some theta or delta task-induced topographic pattern – of P300 appearance – overriding the baseline pattern of the same band). This would mean, if confirmed, that a large fraction of the EEG beyond beta stems from the same set of individual-specific cortical areas, active as long as minimum consciousness is present.

As opposed to the beginnings of our line of research, when tasks were chosen based on neuroanatomical and neuropsychological hypotheses, our criteria were changed by results from the last years. The visual attention task, that became a ‘standard’ in our laboratory, was designed as a simplification of Posner’s tasks [48], [49]. The purpose was exactly to test whether simpler SP distributions and estimated sources, more common across subjects as compared to our first studies, could be observed. It is undeniable that attention is a minimum component process of voluntary activity, especially so in the present task, where location and time of possible target are well known to the subjects. We are currently interested in a “coarse-grained” spectrum of the subjects’ psychological engagement, at least as a first step of use of the currently presented topographic methods, instead of the more fine-grained preconceived functions estimated from Neuroanatomy with which we started this line of research. The reasoning task represents the uppermost level of this spectrum. In the immediate future, we intend to compare the visual attention task with a special ‘resting’ condition, with quiet meditative attention to breathing, eyes opened and fixated, and presentation of the same visual stimuli, but to be ignored. Ideally, we intend to use 256-channel recordings and a free number of ICA components by the required higher capacity computational resources (for easier artifact elimination and preservation of ‘good’ components). More important should be the incorporation of spatial (electrode positions or source locations) into the multiple regression analysis. This would allow for inter-session replication of individual results and, when a satisfactory method of quantification of CDR results in comparable cortical areas across individuals also becomes available, an analogous direct comparison between individuals. A final step of this project will be the study of minimal consciousness state patients. When computing corrected-latency beta averages from the attention task, we obtained preliminary data showing that the main pre-stimulus baseline component appears topographically identical to the main component of an uncontrolled resting state. If high similarity is observed between the meditative resting (and maybe even minimal consciousness state) and attention correlates as well, it would reinforce the idea that the most important aspect of electrical activity is not related to what the subject does, but to the individually idiosyncratic, spontaneously formed functional loops. The implication of this hypothesis to Functional Neurosurgery is that indeed ‘inert’ cortical areas could exist, at least in a temporary mode, whose lesions would have minimal clinical implications, except for the loss of resources to the cortical circuit type of neural plasticity. The major psychophysiological implication of the hypothesis is that most of the neocortex would have a very general, “associative” function (of course still respecting major anatomical-connectional differences such as between frontal and posterior areas), similarly engaged in an equally general process of biological problem solving. This does not explain, but may reduce our astonishment with respect to how organisms of radically divergent neural architecture, such as *octopus vulgaris* with its large “integrative” (non-sensory-motor) lobes, can solve problems in analogous ways to vertebrates.
